# Atherosclerosis in early rheumatoid arthritis: very early endothelial activation and rapid progression of intima media thickness

**DOI:** 10.1186/ar3116

**Published:** 2010-08-16

**Authors:** Anna Södergren, Kjell Karp, Kurt Boman, Catharina Eriksson, Elisabet Lundström, Torgny Smedby, Lisbet Söderlund, Solbritt Rantapää-Dahlqvist, Solveig Wållberg-Jonsson

**Affiliations:** 1 Department of Public Health and Clinical Medicine/Rheumatology, University Hospital, 901 85 Umeå, Sweden; 2 Department of Surgical and Perioperative Sciences, University Hospital, 901 85 Umeå, Sweden; 3 Department of Medicine, Skellefteå Hospital, Lasarettsvägen, 931 86 Skellefteå, Sweden; 4 Department of Clinical Immunology, University Hospital, 901 85 Umeå, Sweden; 5 Department of Rheumatology, Östersund Hospital, Kyrkgatan, 831 83 Östersund, Sweden; 6 Department of Rheumatology, Sunderby Hospital, 971 80 Luleå, Sweden

## Abstract

**Introduction:**

In this study we aimed to investigate whether there are indications of premature atherosclerosis, as measured by endothelial dependent flow-mediated dilation (ED-FMD) and intima media thickness (IMT), in patients with very early RA, and to analyze its relation to biomarkers of endothelial dysfunction, taking inflammation and traditional cardiovascular disease (CVD) risk factors into account.

**Methods:**

Patients from the three northern counties of Sweden diagnosed with early RA are followed in an ongoing prospective study of CVD co-morbidity. Of these, all patients aged ≤60 years were consecutively included in this survey of CVD risk factors (*n *= 79). Forty-four age and sex matched controls were included. IMT of common carotid artery and ED-FMD of brachial artery were measured using ultrasonography. Blood was drawn for analysis of lipids, erythrocyte sedimentation rate (ESR), C-reactive protein (CRP), plasminogen activator inhibitor-1 (PAI-1), tissue plasminogen activator (tPA)-mass, VonWillebrand factor (VWF), soluble intercellular adhesion molecule-1 (sICAM), soluble vascular cell adhesion molecule-1 (sVCAM), sE-selectin, sL-selectin and monocyte chemotactic protein-1 (MCP-1). In a subgroup of 27 RA patients and their controls the ultrasound measurements were reanalysed after 18 months.

**Results:**

There were no significant differences between RA patients and controls in terms of IMT or ED-FMD at the first evaluation. However after 18 months there was a significant increase in the IMT among the patients with RA (*P *< 0.05). Patients with RA had higher levels of VWF, sICAM-1 (*P *< 0.05) and of MCP-1 (*P *= 0.001) compared with controls. In RA, IMT was related to some of the traditional CVD risk factors, tPA-mass, VWF (*P *< 0.01) and MCP-1 and inversely to sL-selectin (*P *< 0.05). In RA, ED-FMD related to sL-selectin (*P *< 0.01). DAS28 at baseline was related to PAI-1, tPA-mass and inversely to sVCAM-1 (*P *< 0.05) and sL-selectin (*P *= 0.001).

**Conclusions:**

We found no signs of atherosclerosis in patients with newly diagnosed RA compared with controls. However, in patients with early RA, IMT and ED-FMD were, to a greater extent than in controls, related to biomarkers known to be associated with endothelial dysfunction and atherosclerosis. After 18 months, IMT had increased significantly in RA patients but not in controls.

## Introduction

Patients with rheumatoid arthritis (RA) have an increased morbidity and mortality due to cardiovascular disease (CVD) [1,2]. Traditional cardiovascular (CV) risk factors cannot fully explain the increase but inflammation has been shown to contribute to the increased CVD among these patients [3-6]. We, and others, have previously shown that patients with RA have premature atherosclerosis as measured by increased intima media thickness (IMT) of the common carotid artery compared with controls [6-9]. Increased IMT, measured by ultrasound, is regarded as an early indicator of overall atherosclerosis [10], and several studies on IMT in the general population have shown a relation between increased IMT and future CV event [11,12]. Among patients with RA, increased IMT has been associated with traditional CV risk factors, including age and sex, markers of inflammation and disease duration [6,7,9]. Almost all of these studies are, however, cross-sectional and comprise patients with long-standing RA.

Endothelial dysfunction, a sign of very early atherosclerosis, can be assessed by impaired endothelial dependent flow-mediated vascular dilation (ED-FMD) of peripheral arteries, measured by ultrasonography [13]. In the general population, ED-FMD was associated with other risk factors for CVD, predictive of a future CV event, and reversible by treatment with cardioprotective drugs [14]. Studies on patients with RA have been small, and nearly all comprised only patient cohorts with longstanding disease [15,16]. There are also several studies on ED-FMD that analyze treatment outcomes in RA [17,18], but only one so far evaluating patients with early RA [19].

In addition to physiological manifestations, the atherosclerotic process appears to be associated with alterations in biomarkers of inflammation, haemostasis and endothelial function. Several studies have investigated different biomarkers, for example, soluble vascular cell adhesion molecule-1 (sVCAM-1), soluble intercellular adhesion molecule-1 (sICAM-1), soluble (s)E-selectin [20,21], monocyte chemotactic protein-1 (MCP-1) [22,23], soluble (s)L-selectin [24], plasminogen activator inhibitor-1 (PAI-1), tissue plasminogen activator (tPA) and VonWillebrand factor (VWF) [21,25] for an association with endothelial dysfunction, atherosclerosis and/or CVD in the general population. We and others have previously shown associations in patients with RA between atherosclerosis and sICAM-1, sE-selectin and PAI-1 [7,26,27] and between endothelial dysfunction and sVCAM-1 and sL-selectin [28]. Other studies on patients with RA have shown an association between IMT and VWF [8] as well as sVCAM-1 [27]. Furthermore, studies have shown higher levels of mass concentrations of tPA and PAI-1 in addition to higher levels of VWF, sVCAM-1, sICAM-1 and sE-selectin in RA patients compared with healthy controls [7,27,29]. However, there are no studies in this respect in patients with early RA. Our hypothesis evaluated in this study was that premature atherosclerosis, and biomarkers of endothelial dysfunction, would be already present in patients with newly diagnosed RA.

From an ongoing prospective case-control study we here present the first cross-sectional baseline data as well as data on progression of atherosclerosis after 18 months. Our primary aim was to investigate whether signs of atherosclerosis, as measured by IMT and ED-FMD, were present in patients with very early RA compared with controls at baseline and after a period of 18 months. The second aim was to identify biomarkers of endothelial activation of particular interest in RA, collected at baseline, that could reflect early atherosclerosis in the context of inflammation.

## Materials and methods

### Patients and controls

This study is part of a continuing structured programme on early RA, using the nationwide Swedish Rheumatoid Arthritis Registry. All eligible patients with newly diagnosed RA [30] and being symptomatic for no longer than 12 months are continuously enrolled into the register. Between 2000 and 2004 all patients under 60 years of age from the three most northern counties of Sweden were consecutively invited to participate in an extended survey on CV morbidity; 79 patients agreed to participate, and were enrolled into this study whilst six patients declined participation, one was pregnant and another was excluded due to advanced breast cancer. Forty-four controls without RA were assembled, of whom 39 were randomly selected from the population register of the same region. In order to include appropriate very young (21-27 years old) controls, three hospital staff and two students were recruited at random. The controls (one control for two patients, but in nine cases one control per patient) were matched for age (± 5 years) and sex. All individuals gave their written consent in accordance with the Declaration of Helsinki. The study was approved by the Regional ethics committee of Umeå University, Umeå, Sweden.

### Physical examination and surveys

All patients were examined clinically at inclusion into the study. The number of swollen and tender joints (28 joint count) and patient's global assessment were registered and a disease activity score (DAS28) including erythrocyte sedimentation rate (ESR) calculated [31]. All participants were requested to complete a health assessment questionnaire (HAQ) [32], a survey on co-morbidity, including any previous CVD event as defined by acute myocardial infarction, stroke or coronary artery bypass surgery, and a survey of CVD risk factors and life style [33]. Body mass index (BMI) was calculated. Medical treatments at the time of the first ultrasound measurements are presented in Table 1.

**Table 1:** Demographic data and traditional CVD risk factors in patients with early RA and age- and sex-matched controls

	RA (*n *= 79)	Controls (*n *= 44)
Women	64 (80%)	34 (77%)
Age, years	46.4 (10.7)	47.7 (11.1)
Systolic blood pressure, mmHg	123.6 (14.3)*	117.4 (11.0)
Diastolic blood pressure, mmHg	77.3 (8.6)	76.3 (8.3)
Heart rate, beats per minute	72.3 (10.5)	67.9 (9.4)
Cholesterol, mmol/L	5.40 (0.96)	5.39 (1.15)
Triglycerides, mmol/L	1.25 (0.49)*	1.06 (0.38)
HDL, mmol/L	1.52 (0.53)	1.44 (0.39)
BMI, kg/m^2^	25.7 (4.0)	25.1 (4.9)
Smoking, years	13.75 (14.32)*	6.96 (10.33)
Oral snuff, years	3.12 (8.01)	2.57 (7.39)
Diabetes mellitus	5 (6%)	1 (2%)
Previous CVD event	5 (6%)	1 (2%)
Postmenopausal	25 (42%)	16 (47%)
University education	23 (30%)	20 (46%)
Familial history of CVD	17 (24%)	7 (18%)
Treatment with NSAIDs	35 (50%)	2 (5%)
Treatment with coxibs	9 (13%)	1 (2%)
Treatment with statins	2 (3%)	1 (2%)
Treatment with corticosteroids	26 (38%)	---
Treatment with DMARDs	74 (97%)	---

Data are expressed as mean value (standard deviation) or number of individuals (percentage for whom data are given).

* *P *< 0.05, ** *P *< 0.01, *** *P *< 0.001.

The DMARDS patients received were 41 methotrexate, 12 sulphasalazine, 1 oral gold, 8 were receiving a combination of methotrexate and hydroxychloroquine phosphate, 4 a combination of methotrexate and sulphasalazine, 2 had a combination of methotrexate, sulphasalazine and hydroxychloroquine phosphate and 1 had a combination of methotrexate, sulphasalazine and etanercept.

BMI, body mass index; CVD, cardiovascular disease; DMARDs, disease-modifying antirheumatic drugs; HDL, high-density lipoproteins; NSAIDs, non-steroidal anti-inflammatory drugs; RA, rheumatoid arthritis.

### Ultrasound investigations

Ultrasound examinations were performed as soon as possible after the first symptoms of RA, and no longer than 12 months after diagnosis (mean 16.2 ± 6.6 months after the first symptom). All examinations were performed by the same experienced investigator with the individuals in a supine position in a quiet, temperature-controlled room. A Sequoia 512 ultrasound system (Siemens (Acuson) Corp, UpplandsVäsby, Sweden) was used with a 15L8 transducer for brachial artery and a 8L5 transducer for carotid artery studies. All investigations were digitally stored for analyses, which were performed by a single observer (EL, intraobserver variability r = 0.988) on a Sequoia 512.

R-wave triggered end diastolic right brachial artery longitudinal images proximal to the ante cubital fossa were recorded at baseline after 15 minutes of supine rest. Transducer position was carefully noted for subsequent investigations. For ED-FMD R-wave triggered images were stored for 90 seconds following ischaemia. The ischaemia was induced using a cuff on the forearm inflated 20 mmHg above the systolic blood pressure for five minutes with additional lower arm muscular work by repetitively squeezing a small ball during the last minute of ischaemia. Maximal brachial artery diameter was calculated for both conditions as a mean of three measurements. ED-FMD was calculated as a percentage of the baseline diameter.

Carotid artery studies were performed with the individual in the supine position with the neck extended and the chin turned away from the side being examined. The right common carotid artery proximal to the bulb was imaged in multiple longitudinal planes for the best resolution of the IMT of the far wall. The IMT was obtained manually tracing the intima-media in the far wall of the artery for a distance of approximately 10 mm. Measurements were performed on three end diastolic images and averaged.

In a subgroup of 27 patients with RA and their controls, the ultrasound measurements were retaken 18 months (± 2 weeks) after the first ultrasound investigation.

### Blood sampling

In the present study all patients and controls donated a blood sample at the time of the first ultrasound measurement. This was separated into plasma, serum and buffy coat and stored at -80°C. After thawing, sVCAM-1 (ng/mL), sICAM-1 (ng/mL), sL-Selectin (ng/mL) and sE-Selectin (ng/mL) were measured on serum using ELISA (R&D Systems, Abingdon, UK) and MCP-1 (pg/mL) using ELISA (HyCult Biotechnology, Uden, The Netherlands). Mass concentrations of PAI-1 (μg/L) and tPA (μg/L) were measured on plasma using ELISA (Trinity Biotech Inc, Bray, Ireland) and VWF (%) using ELISA as previously described [34]. The presence of antibodies against cyclic citrullinated peptide (anti-CCP) in serum was detected using the Diastat kit (Axis-Shield Diagnostics, Dundee, UK) with a cut-off value of 5 units/mL. Blood was also drawn after an overnight fast for analysis of blood lipids: cholesterol (mmol/L), high-density lipoproteins (HDL; mmol/L) and triglycerides (mmol/L) were measured by routine methods at each of the hospitals. When a diagnosis of RA was confirmed, rheumatoid factor (RF) and soluble C-reactive protein (CRP; mg/L) were measured according to routine methods at each of the hospitals and ESR (mm/h) was measured according to the Westergren method. ESR and CRP were also measured every three months thereafter. CRP of 10 mg/L or less was set at the cut-off level, 10 mg/L, in the statistical calculations. Whenever several analyses of DAS28, CRP or ESR were performed the assessment closest to the ultrasound measurement was used in the statistical analyses, that is, with a time lag that was at most six weeks but in most cases much shorter.

### Statistics

Differences in variables between patients with RA and matched controls were analyzed using simple conditional logistic regression analyses. Comparisons within the RA group were performed using the Mann-Whitney U-test or, for comparsions over time, the Wilcoxon paired test. Simple and multiple linear regression analyses were used to identify variables associated with ED-FMD, IMT and the biomarkers. Simple linear regression (variables with *P *< 0.05) together with clinical assumptions, determined which co-variates were included in the multiple linear regression models. To check the assumption of linearity, continuous variables were also categorised according to their tertiles and included in a regression model, but all variables were considered as linear. Based on results from previous publications, calculations showed that a sample size of 26 in each group would render 95% power to detect a difference in IMT of 0.1 ± 0.1 mm between the groups. In some of the descriptive statistics there are occasionally missing values due to missing information, these missing values can be regarded as random. *P *< 0.05 were considered statistically significant. All calculations were made using SPSS 15.0 (SPSS Inc, Chicago, IL, USA).

## Results

### Baseline data

Data on demographics and traditional CV risk factors are given in Table 1. The patients with RA had significantly higher systolic blood pressure, higher levels of triglycerides and more years of smoking compared with the controls at baseline.

There were no significant differences between patients and controls regarding any of the ultrasound measurements at baseline (Table 2).

**Table 2:** Baseline measurements of intima media thickness and flow mediated dialtation in patients with early RA and age- and sex-matched healthy controls

	RA (*n *= 79)	Controls (*n *= 44)
Intima media thickness, mm	0.52 (0.13)	0.55 (0.15)
Baseline diameter of a brachialis, mm	3.5 (0.07)	3.5 (0.05)
Endothelium-dependent flow mediated vasodilatation, %	108.9 (4.7)	107.2 (4.6)

Data is given as mean value (standard deviation).

RA, rheumatoid arthritis.

Fifty-five out of 78 (71%) patients were RF seropositive and 50 of 78 (64%) had anti-CCP antibodies. Data on inflammation in patients with RA are given in Table 3. Compared with controls, patients with RA had significantly higher levels of, sICAM-1, MCP-1 and VWF, with a tendency also for sVCAM-1 to be higher (*P *= 0.059; Table 3). The statistically significant differences in VWF, sICAM-1 and MCP-1 levels remained after adjustment for sex, age, smoking, systolic blood pressure or any of the lipids (data not shown).

**Table 3:** Endothelial biomarkers and markers of inflammation in patients with early RA and age- and sex-matched healthy controls

	RA (*n *= 79)	Controls (*n *= 44)
sVCAM-1, ng/mL	742.7 (237.9) (*)	631.4 (173.2)
sICAM-1, ng/mL	354.1 (131.8)*	290.6 (66.9)
sE-selectin, ng/mL	51.8 (19.2)	53.8 (16.7)
sL-selectin, ng/mL	1252.0 (306.2)	1279.7 (273.2)
MCP-1, pg/mL	1945.6 (936.9)***	1125.1 (475.5)
PAI-1, μg/L	64.1 (37.3)	59.1 (28.7)
tPA-mass, μg/L	7.17 (3.04)	8.06 (4.32)
VWF, %	203.1 (74.1)*	161.8 (61.2)
ESR, mm/h	18.6 (16.7)	---
CRP, mg/L	13.6 (13.9)	---
DAS28	3.62 (1.4)	---
HAQ	0.65 (0.6)	---

Data is given as mean value (standard deviation).

(*) = 0.059, * *P *< 0.05, ** *P *< 0.01, *** *P *< 0.001.

CRP, C-reactive protein; DAS28, disease activity score; ESR, erythrocyte sedimentation rate; MCP-1, monocyte chemotactic protein-1; HAQ, health assessment questionnaire; PAI-1, plasminogen activator inhibitor-1; tPA, tissue plasminogen activator; RA, rheumatoid arthritis; sICAM-1, soluble intercellular adhesion molecule-1; sVCAM-1, soluble vascular cell adhesion molecule-1; VWF, VonWillebrand factor.

In the RA patients, IMT at baseline was significantly related to some of the traditional CVD risk factors when analyzed in simple linear regression models (Table 4). Regarding endothelial biomarkers, IMT was significantly related to tPA-mass, VWF, sL-selectin and MCP-1 and ED-FMD was significantly related to PAI-1 and sL-selectin (Table 4).

**Table 4:** Simple linear regression models of associations between some of the traditional risk factors for CVD, markers of inflammation and endothelial biomarkers and IMT or ED-FMD in 79 patients with early RA

	IMT		ED-FMD	
		
	β	*P*	β	*P*
Sex, female	-1.02/+	0.005	3.82/+	0.002
Age	0.001/year	<0.001	- 0.178/year	<0.001
Systolic BP	0.030/mmHg	0.001^†^		ns
Cholesterol	0.004/mmol L^-1^	0.027^†^		ns
Smoking, years	0.029/year	0.005^†^		ns
Snuff, years	0.043/year	0.027^†^		ns
PAI-1		ns	-0.026/μg L^-1^	0.064^**†**^
tPA-mass	73.0/μg L^-1^	0.005^†^		ns
VWF	5.914/%	0.003^†^		ns
sL-selectin	-1.058/ng mL^-1^	0.026^†^	4.961/ng mL^-1^	0.004^†^
MCP-1	4.650/pg mL^-1^	0.002		ns

^† ^Not significant after adjustment for sex and/or age.

BP, blood pressure; CVD, cardiovascular disease; ED-FMD, endothelial dependent flow mediated dilatation; IMT, intima media thickness; MCP-1, monocyte chemotactic protein-1; ns, Not significant; PAI-1, plasminogen activator inhibitor-1; RA, rheumatoid arthritis; tPA, tissue plasminogen activator; VWF, VonWillebrand factor.

Among the controls, IMT was significantly related to systolic blood pressure (β = 0.07, *P *< 0.001), age (β = 0.08, *P *< 0.001), triglyceride level (β = 2.3, *P *< 0.001), cholesterol level (β = 0.6, *P *< 0.001), HDL level (β = -1.6, *P *< 0.01), BMI (β = 0.1, *P *< 0.05) and female sex (β = -2.1, *P *< 0.001), but regarding biomarkers only to tPA-mass (β = 0.2, *P *< 0.01) and sL-selectin (β = -1.8, *P *< 0.05). In the controls ED-FMD was significantly related only to MCP-1 (β = -33.4, *P *< 0.05). There were no other statistically significant relations between ultrasound measurements and risk factors in patients or controls at baseline (data not shown).

There was no relation between any of the disease activity measurements, that is, DAS28, HAQ, ESR and CRP level, and the ultrasound measurements in the RA patients at the time of the baseline investigation. Nor were there any relations between any of the disease-modifying anti-rheumatic drugs (DMARDs) and any of the variables studied (data not shown).

Several of the endothelial biomarkers were related to traditional CV risk factors as well as to markers of inflammation in the patient cohort (Table 5). Furthermore, there was a strong relation between MCP-1 and tPA-mass (β = 13.813, *P *< 0.001) in patients with RA.

**Table 5:** Simple linear regression models of associations between endothelial biomarkers and sex, age, blood pressure or DAS28 in 79 patients with early RA

	Sex (female)		Age		Systolic BP		DAS28	
				
	β	*P*	β	*P*	β	*P*	β	*P*
PAI-1	-22.1/+	0.034	0.84/year	0.033	0.58/mmHg	0.045^†^	6.40/unit	0.048^†^
tPA-mass	-2.2/+	0.008	0.11/year	0.001	0.064/mmHg	0.008^†^	0.50/unit	0.045^†^
VWF		ns	3.36/year	<0.001	1.53/mmHg	0.012^†^		ns
sVCAM-1		ns		ns		ns	-48.1/unit	0.020
sICAM-1		ns	2.76/year	0.048		ns		ns
sE-selectin		ns		ns		ns	2.99/unit	0.073
sL-selectin	267.2/+	0.002	-14.4/year	<0.001	-6.9/mmHg	0.008^†^	-63.8/unit	0.001
MCP-1		ns	21.3/year	0.031	15.5/mmHg	0.046^†^		ns

^†^Not significant after adjustment for sex and/or age.

BP, blood pressure; DAS28, disease activity score; MCP-1, monocyte chemotactic protein-1; ns, not significant; PAI-1, plasminogen activator inhibitor-1; RA, rheumatoid arthritis; sICAM-1, soluble intercellular adhesion molecule-1; sVCAM-1, soluble vascular cell adhesion molecule-1; tPA, tissue plasminogen activator; VWF, VonWillebrand factor.

Sex and, in particular age, were strong predictors for some of the endothelial biomarkers. In a multiple linear regression model taking into account solely the predictive value of the endothelial biomarkers for increased IMT in patients with RA, VWF and MCP-1 were the best predictors (Table 6, model 1). When the data were adjusted for disease activity, the same relations were still apparent (Table 6, model 2).

**Table 6:** Multiple linear regression models of some of the inflammatory markers, and endothelial biomarkers with IMT as response variable in 79 patients with early RA

	Model 1			Model 2		
		
	β	95% CI	*P*	β	95% CI	*P*
VWF	4.420	0.617; 8.223	0.023	3.946	0.334; 7.557	0.033
tPA-mass	0.069	-0.027; 0.164	0.156	0.026	-0.070; 0.122	0.588
sICAM-1	0.200	-1.950; 2.349	0.854	0.616	-1.461; 2.693	0.556
sL-selectin	-0.719	-1.585; 0.147	0.102	-0.759	-1.631; 0.112	0.087
MCP-1	3.117	-0.022; 6.256	0.052	3.406	0.407; 6.405	0.027
ESR				-0.007	-0.023; 0.009	0.384

ESR, erythrocyte sedimentation rate; CI, confidence interval; IMT, intima media thickness; MCP-1, monocyte chemotactic protein-1; RA, rheumatoid arthritis; sICAM-1, soluble intercellular adhesion molecule-1; tPA, tissue plasminogen activator; VWF, VonWillebrand factor.

In the same model, but with ED-FMD in RA as the response variable, sL-selectin was a highly significant predictor (Table 7, model 1). This was true also in a model where only the variables that were significant in simple regression (*i.e. *PAI-1 and sL-selectin) were tested (data not shown). Again, when the data was adjusted for disease activity, this relation was maintained (Table 7, model 2). These predictive values for the biomarkers remained after further adjustment for systolic blood pressure and smoking (data not shown).

**Table 7:** Multiple linear regression models of inflammatory markers, and endothelial biomarkers with ED-FMD as response variable in 79 patients with early RA

	Model 1			Model 2		
		
	β	95% CI	*P*	β	95% CI	*P*
VWF	6.594	-8.410; 21.598	0.384	5.099	-9.924; 20.122	0.501
tPA-mass	-0.339	-0.716; 0.038	0.077	-0.366	-0.766; 0.034	0.072
sICAM-1	-2.682	-11.164; 5.799	0.530	-3.201	-11.840; 5.439	0.462
sL-selectin	5.125	1.708; 8.542	0.004	6.406	2.780; 10.033	0.001
MCP-1	4.476	-7.908; 16.860	0.474	6.387	-6.087; 18.862	0.311
ESR				0.048	-0.018; 0.114	0.114

CI, confidence interval; ED-FMD, endothelial dependent flow mediated dilatation; ESR, erythrocyte sedimentation rate; MCP-1, monocyte chemotactic protein-1; RA, rheumatoid arthritis; sICAM-1, soluble intercellular adhesion molecule-1; tPA, tissue plasminogen activator; VWF, VonWillebrand factor.

When the same models were tested for the controls, tPA-mass was the best predictor for IMT (β = 0.18, *P *< 0.01) and MCP-1 for ED-FMD (β = -37.85, *P *< 0.05).

### Ultrasound measurements at 18 months

In the subgroup that was re-evaluated 18 months after the first ultrasound measurement, the IMT had increased significantly among the RA patients to (mean (standard deviation) at 18 months) 0.57 ((0.15); *P *< 0.05). Among the controls the IMT after 18 months was 0.54 ((0.13); not significant). The endothelium-dependent vasodilatation did not change significantly neither among the RA patients nor among the controls (106.9 (5.2) and 107.5 (4.4), respectively; not significant). There were no statistically significant differences in the difference in changes of the measurements, that is, delta values, between RA patients and controls after 18 months (data not shown).

## Discussion

We found no impairment of the endothelial function, measured as ED-FMD, among patients with newly diagnosed RA compared with controls. There have been, to the best of our knowledge, only two previous studies on ED-FMD in newly diagnosed RA patients [19,35]. In those studies, patients with RA had a blunted endothelium-dependent vasodilatation. However, our study comprises four to eight times more patients with RA than those studies, which might in part explain the different results.

There was no significant difference in IMT in patients with early RA compared with controls. There are few studies evaluating atherosclerosis in newly diagnosed RA patients. In two recent studies, RA patients had an increased IMT compared with healthy controls [36,37]. Those studies comprised older patients with higher inflammatory activity than those in the present study. Moreover, there were several other methodological differences compared with the present study that might explain the different results. Also, in another recent study, evaluating coronary calcification in early RA, there were no difference in atherosclerosis compared with controls [38]. However, in a tentative follow-up after 18 months we found a significant increase in the IMT among patients with RA while there was no equivalent significant change in the control group. This increase was significant although all patients with RA were treated with DMARDs and had a low inflammatory activity. In longstanding RA we have previously demonstrated an increased IMT [7], a finding later confirmed by other investigators [6,8]. Also in patients with longstanding RA an increase in IMT has been shown even though efficient treatment with anti-TNF therapy [39]. One recent epidemiological study showed an increased incidence of CVD, as measured by myocardial infarction, in patients with RA during the two years prior to diagnosis [40]. The authors concluded that their results could imply the presence of inflammatory-triggered atherosclerosis before overt symptoms and diagnosis of RA.

We also wanted to identify endothelial biomarkers reflecting the early atherosclerotic process in RA. Thus, we evaluated the involvement of adhesion molecules, MCP-1 and endothelially produced haemostatic factors in endothelial dysfunction and very early atherosclerosis, as measured by ED-FMD and IMT, respectively. Simple regression analyses revealed that decreased sL-selectin was strongly related to a lower ED-FMD in RA and associated with an increased IMT in both RA and controls. A lower level of sL-selectin has been associated with CVD in the general population [24]. L-selectin is expressed on leucocytes and is also known to be involved in the initial leukocyte rolling on activated endothelium [24]. L-selectin has been shown to be downregulated during chronic inflammation. We found a strong inverse relation between sL-selectin and DAS28, which reflects the current inflammatory status in RA. In multiple linear regression models, evaluating the impact of endothelial biomarkers on endothelial function and adjusting for inflammation, sL-selectin was still strongly related to ED-FMD and also close to significantly related to IMT. This implies a durable relation between sL-selectin and endothelial function in early RA, a relation that does not seem to be mediated simply via inflammatory disease activity. This is a new finding that warrants further investigation of pathophysiological mechanisms beyond the inflammatory pathway.

Regarding the other adhesion molecules, sVCAM-1 and sICAM-1 showed higher levels in RA than in controls; however, we did not find any independent relations with any of the ultrasound measurements among our relatively young patients with early RA. Although, in agreement with the findings of others, some of the adhesion molecules showed relations to measures of inflammation [27]. In previous studies on older patients with long-standing RA, several of these markers correlated with IMT [26,27]. The inflammatory status of the present patient cohort was, however, low and of short duration, leading to less stress on the endothelium.

MCP-1 was found to be a strong and independent predictor of an increased IMT. This chemoattractant for monocytes is secreted by activated endothelial cells and has been suggested to be a marker of endothelial inflammation, the first step in atherosclerosis [23]. Moreover, MCP-1 has been shown to promote plaque growth, to be increased in acute CVD in the general population [23] and to be correlated with IMT in uraemic patients [22]. In the present study MCP-1 was related to tPA-mass, which in turn was associated with markers of inflammation both here and in other studies [41]. However, when adjusting for inflammation and other endothelial biomarkers, the impact of MCP-1 on IMT was even more significant. We have previously found increased MCP-1 to precede the diagnosis of RA in anti-CCP or RF-positive individuals [42]. Previously, a more atherogenic lipid profile, suggested to modify the inflammatory reaction, has been shown to antedate the diagnosis of RA by at least 10 years [43]. Taken together, these findings are suggestive of a very early endothelial activation in RA, possibly already present before diagnosis.

Regarding the haemostatic factors, increased tPA-mass was related to a higher IMT. There was also a tendency towards a significant relation between increased PAI-1-mass and impaired ED-FMD. Increased values for PAI-1 and tPA mass have been shown to be associated with an increased risk of CVD in the general population [21,25] as well as in patients with RA [26]. tPA is produced endothelially and an increase in this biomarker might be another reflection of an endothelial activation [21,25]. In this study, there was a relation between tPA-mass and DAS28 in patients with RA. Increasing PAI-1-mass was also related to markers of inflammation. PAI-1 is an acute-phase reactant and this finding is consistent with our previous study in which PAI-1 was increased in RA patients compared with controls [7]. Both tPA and PAI-1 have also been shown to be associated with inflammation in patients with diabetes mellitus [41]. An increase in these biomarkers leads to a prothrombotic state [21]. Furthermore, our finding that the level of VWF, another endothelially produced haemostatic factor, was independently related to the IMT, is in accordance with other reports [8,21]. This relation also remained when adjusted for ESR. Taken together, our findings implicate a procoagulant state among our RA patients with newly diagnosed RA.

It appears, as a result of this study, that sL-selectin, being involved in the initial leukocyte rolling process, is a strong predictor of the early endothelial activation in RA, as measured by ED-FMD, whereas MCP-1 and VWF are indicators of increasing IMT, a later phase of the early atherosclerotic process. Corresponding results were not found in the control group.

In the RA group, the IMT was higher among men than females, and IMT also increased with age, higher systolic blood pressure, higher cholesterol and a longer period of smoking or using oral snuff. Also, there was a decrease in ED-FMD in men compared with women, ED-FMD decreased with increasing age and increasing systolic blood pressure. All of these findings are in line with studies on the general population [11,12,14] or patients with established RA [4].

Our ongoing study is one of the first to investigate the ED-FMD and IMT prospectively among newly diagnosed patients with RA. A strength of the study is that in northern Sweden nearly all patients with newly diagnosed RA are included in a structured follow up. All patients aged 60 years or younger were asked to participate in this study within 12 months of their diagnosis. As our main interest is to evaluate the impact of inflammation and endothelial activation on the early atherosclerotic process prospectively over a long time, a young cohort comprising patients with very early RA will be less affected by obscuring generalised atherosclerosis [42] and by other comorbidities and mortality in the coming decades. Moreover, as inflammation seems to be relatively more important for the atherosclerotic progression in younger patients [4] this cohort should, however, be ideal for unmasking any difference prospectively. Although it may have rendered it more difficult to determine baseline differences in RA vs. controls, the design allows repeated analyses in a comparably large cohort. The first follow-up only 18 months after the initial ultrasound measurement could be accomplished in only a subgroup. Nevertheless, this provided a unique opportunity to investigate rapid changes in the measurements of atherosclerosis among patients with RA. A complete follow-up in all RA patients and controls five years after the first ultrasound investigation is in progress. Another strength of this study was that the same person (EL) undertook all of the ultrasound measurements, thereby eliminating interpersonal variation. A limitation is that there was only one control per two RA patients, which could decrease the statistical power in the control group. Both the control group and, in particular, the RA-group are, however, larger than in most previous studies on RA using this technique. Furthermore, our main focus regarding associations between physiological measures and biomarkers was in the larger RA-group. Another limitation could be that all individuals were already on and continued their medications, including DMARDs, corticosteroids, non-steroidal anti-inflammatory drugs and coxibs throughout the ultrasound measurement, due to the fact that this work is part of a prospective observational study. Medications at the first ultrasound investigation, or up until the first follow-up, had however no statistically significant influence on the results.

Taken together, our results point towards an ongoing endothelial activation among patients with early RA. This endothelial activation was not translated into endothelial dysfunction, as measured by ED-FMD. Changes of ED-FMD can be rapid and could have reflected a high inflammatory activity if present. The variability of IMT is complex and therefore more inert to temporary changes. A more sensitive physiological method would possibly have been able to uncover a relation. However, there were differences in the levels of biomarkers, indicating endothelial activation in early RA to be related to the disease process, yet independent of acute-phase reaction. Also, after 18 months this had been translated into a significant increase of atherosclerosis as measured by IMT, not evident among the controls. Furthermore, more biomarkers were related to the physiological measurements within the RA patient cohort than among the controls, while the controls exhibited more relations between the physiological measurements and traditional CVD risk factors. This supports the hypothesis that the disease process in RA is involved in early endothelial activation and atherosclerosis, in a way distinguished from the general population. This possibly indicates that the endothelial activating process also has a different course compared with that leading to joint destruction in RA. In previous studies of patients with long-standing RA, in whom endothelial activation had continued for several years, the IMT was consistently shown to be increased compared with controls [6-9].

## Conclusions

In conclusion, we found no signs of increased atherosclerosis, as assessed by IMT and ED-FMD measurements, among patients with newly diagnosed RA compared with healthy controls. However, both IMT and ED-FMD were related to several biomarkers of endothelial activation, mainly to MCP-1 and sL-selectin, respectively. This may implicate an endothelial activation occurring very early in the course of RA preceding signs of endothelial dysfunction and premature atherosclerosis, as measured by ED-FMD and IMT. Considering the current state of knowledge, the necessity of optimising prevention and treatment of CVD in patients with RA must be emphasised.

## Abbreviations

anti-CCP: antibodies against cyclic citrullinated peptide; BMI: Body mass index; CRP: C-reactive protein; CV: cardiovascular; CVD: cardiovascular disease; DAS28: disease activity score; DMARD: disease-modifying anti-rheumatic drug; ED-FMD: endothelial-dependent flow-mediated dilation; ELISA: enzyme-linked immunosorbent assay; ESR: erythrocyte sedimentation rate; HAQ: health assessment questionnaire; HDL: high-density lipoprotein; IMT: intima media thickness; MCP-1: monocyte chemotactic protein-1; PAI-1: plasminogen activator inhibitor-1; RA: rheumatoid arthritis; RF: rheumatoid factor; sICAM-1: soluble intercellular adhesion molecule-1; sVCAM-1: soluble vascular cell adhesion molecule-1; TNF: tumour necrosis factor; tPA: tissue plasminogen activator; VWF: VonWillebrand factor.

## Competing interests

The authors declare that they have no competing interests.

## Authors' contributions

AS participated in the design of the study, acquired data, performed the statistical analysis and drafted the manuscript. KK participated in the design of the study and the evaluation of the ultrasound data. KB participated in the design of the study and accomplished the haemostatic data. CE carried out the immunoassays. EL carried out the ultrasound measurements. TS and LS facilitated the acquisition of clinical data. SRD participated in the design of the study and contributed to a great extent to the discussion. SWJ conceived of the study, and participated in its design and coordination and helped to draft the manuscript. All authors were contributing in discussions and read and approved the final manuscript.
